# Assessing TEG6S reliability between devices and across multiple time points: A prospective thromboelastography validation study

**DOI:** 10.1038/s41598-020-63964-y

**Published:** 2020-04-27

**Authors:** Patryck Lloyd-Donald, Leonid Churilov, Brandon Cheong, Rinaldo Bellomo, Peter R. McCall, Johan Mårtensson, Neil Glassford, Laurence Weinberg

**Affiliations:** 1 Department of Anaesthesia, Austin Hospital, 145 Studley Rd, Heidelberg, 3084 Victoria Australia; 2 Department of Medicine, Austin Health, Melbourne Medical School, University of Melbourne, 245 Burgundy St, Heidelberg, 3084 Victoria Australia; 3 Department of Intensive Care, Austin Hospital, 145 Studley Rd, Heidelberg, 3084 Victoria Australia; 4 Department of Perioperative Medicine and Intensive Care Medicine, Karolinska University Hospital, Solna, and Department of Physiology and Pharmacology, Karolinska Institutet, SE-171 77 Stockholm, Sweden; 5 Department of Surgery, Austin Health, University of Melbourne, 3010 Victoria, Australia

**Keywords:** Diagnosis, Medical research

## Abstract

The TEG6S is a novel haemostasis analyser utilising resonance technology. It offers potentially greater coagulation information and ease of use, however has not been independently validated in a clinical setting. We aimed to determine if the TEG6S is reliable between devices and across time points. We performed a prospective observational study with ethical approval. For interdevice reliability, we performed simultaneous analysis on two TEG6S devices on 25 adult ICU patients. For time point reliability, we performed repeated sampling across five different time points on 15 adult participants. Blood was collected with informed consent, or as standard care, before four-channel citrated kaolin analysis. We observed almost perfect interdevice reliability across all TEG parameters. The Lin’s concordance correlation coefficients (95% CI, major axis regression slope, intercept) were R-time: 0.96 (0.92–0.99, 0.88, 0.57); K-time: 0.93 (0.87–0.98, 1.07, 0.00); Alpha Angle: 0.87 (0.78–0.96, 1.20, −14.10); Maximum Amplitude: 0.99 (0.98–0.99, 1.02, −1.38); Clot Lysis: 0.89 (0.82–0.97, 1.20, 0.07). Additionally, we observed moderate-to-high reliability across time points. Demonstrating almost perfect agreement across different devices and moderate-to-high reliability across multiple time points, suggests the TEG6S platform can be used with haemostatic accuracy and generalisability. This has potentially significant implications for clinical practice and multi-site research programs.

## Introduction

Thromboelastography (TEG) is a method of assessing coagulation using viscoelastic properties of blood in order to provide a global assessment of coagulation compared to more traditional laboratory coagulation tests^1^. Internationally, there are two main TEG platforms used in clinical practice. The older TEG5000® (Haemonetics, Massachusetts, USA) system measures the shear elasticity of a coagulating sample using an electrical-mechanical transducer of movement of torsion wire connected to the suspended pin. Each TEG assay is performed manually which requires lengthy preparation and calibrated pipetting^2^. The newer TEG6S® platform measures clot viscoelasticity throughout the coagulation process by using resonance technology, exposing the blood sample to a fixed vibration frequency^3,4^. Using LED illumination, an infrared detector measures vertical motion of the coagulating blood meniscus^5^. The changes in resonance when the blood coagulates are recognised by the TEG6S analyser and converted to a graphical image^4,6^. This process is fully automated, which eliminates the need for manual pipetting, simplifying and standardising the process. From a single blood sample, the TEG6S also enables multiple assays (citrated kaolin, heparinase kaolin, rapid TEGs and functional fibrinogen) to be performed simultaneously, in contrast to the TEG5000 system that only permits a single assay to be run at a given time^7,8^.

Having previously determined the agreement between the existing TEG5000 and TEG6S devices^9^, this study aimed to assess reliability and clinical interchangeability of the TEG6S coagulation parameters when assessed by different TEG6S devices (i.e. interdevice agreement). Further we assessed device reliability across multiple time points (test re-test agreement). The aims of this study were to independently validate the new TEG6S platform for clinical and research reliability.

## Materials and methods

The Austin Health Human Research Ethics Committee approved this study (HREC number: 05006/2013) and granted a waiver of consent for patients who required TEG analysis as part of routine clinical care. Volunteers were recruited through an advertisement leaflet in the departments of anaesthesia and intensive care, and all volunteers provided informed consent. The study was registered with the Australian New Zealand Clinical Trials Registry (ACTRN no: 12616001698460). All experiments were performed in accordance with relevant guidelines and regulations.

### Study design

We conducted a prospective, observational study at a tertiary metropolitan hospital with a dedicated cardiac and hepatobiliary-pancreatic surgical service, including liver transplantation. All patients were recruited at the Austin Hospital, Melbourne, Australia between July 2015 and January 2016.

The primary outcome was interdevice agreement. We first hypothesised that there would be almost perfect interdevice agreement (Lin’s concordance coefficient above 0.8) in TEG results when a sample of blood drawn by a single operator from a single ICU patient was simultaneously tested on two TEG6S devices. The automation provided by the TEG6S platform simplifies and standardises the process, ensuring accuracy and a generalisable clinical picture of haemostasis across centres that use the same clinical platform. Interdevice reliability in turn can enhance multisite collaborative research capability.

Having established good interdevice reliability in Hypothesis 1, we treated the devices as interchangeable and performed an additional, secondary, exploratory analysis to evaluate time point (test re-test). Given that the TEG6S platform requires whole blood to be collected in a citrated vial, this allows for more time, or a planned delay between the blood draw and the initiation of the test. This also has important benefits for clinical research where a delay in testing may be necessary for pragmatic reasons. Accordingly, we tested the hypothesis that there would be high agreement in the TEG parameters if a sample of blood was simultaneously tested across preset time points of 0 minutes (immediately after collection), and then again at 15, 60, 120 and 180 minutes (i.e. test re-test reliability).

### Eligibility

For interdevice reliability we included adult patients (age >18 years) undergoing surgery or critically ill patients in our ICU with an existing arterial line *in-situ* and who required a TEG test as part of standard care. In order to assess TEG6S performance on these patients, no exclusion criteria were imposed. Similarly, for time point reliability, samples were collected from surgical and ICU patients who had an existing arterial line *in-situ* and required a TEG as part of standard care. A deliberately heterogeneous sample cohort was selected at random via purposive sampling to reflect a broad mix of age and gender in the population of interest. No exclusions were imposed based on anticoagulation or known coagulopathy. All samples were taken from ICU patients within 72 hours of their hospital admission.

Volunteers were all adults (age >18 years) and were selected via a purposive sampling method with no exclusions placed based on anticoagulation, known coagulopathy or otherwise. Patients (interdevice) and volunteers (time point) were only enrolled once.

### Thromboelastography analysis

As previously described, the TEG6S global haemostasis system provides four assays in one multi-channel cartridge, with each assay measuring a specific component of the coagulation process^9^. The first assay is citrated kaolin (CK), containing the coagulation activator, kaolin, providing clot reaction time (R time: the time from the start of analysis until thrombus amplitude reaches 2 mm), clot kinetics (K time: the time from thrombus amplitude reaching 2 mm until amplitude reaches 20 mm), thrombus generation angle (alpha angle: slope of a tangent line from the tracing at the midpoint between the R and the K time), thrombus maximum amplitude (MA: absolute thrombus strength) and percentage of fibrinolysis (LY30%). The second assay is Rapid-TEG assay (RT): containing tissue factor and kaolin, providing R time, K time, alpha angle, MA, LY30% and an activated clotting time (ACT: measuring initiation of clotting phase). The third assay is heparinase kaolin assay (HK): containing kaolin and heparin-neutralising enzyme, providing R time, K time, alpha angle and MA. The final assay is functional fibrinogen (FF): providing maximum amplitude (MA) based on fibrinogen contribution to the thrombus, and functional fibrinogen level (an estimation of plasma fibrinogen level). All samples from all patients were analysed using the same 4-channel TEG method. Platelet-mapping TEG was not performed. Each of the TEG devices were calibrated appropriately with biological quality controls. Treating clinicians were not blinded to the results of the patient group as this was part of standard care. Operators were neither blinded to either TEG results or clinical information of the patients.

*Sampling and data-collection* We sampled patient blood from an existing arterial line. Five milliliters of blood was aspirated and discarded. A further 3.5 mL was drawn into a single vacuette vial (Greiner Bio-One, North Carolina, USA) containing 3.2% citrate solution. Blood was obtained from volunteers via peripherally inserted 20-gauge butterfly venepuncture needle as per standard practice. All samples were stored upright at room temperature (20–25 °C). Interdevice samples were analysed after between 15 and 20 minutes, as recommended. Time point (test re-test) samples were analysed at 0, 15, 60, 120 and 180 minutes. For pragmatic reasons, 5 TEG devices were used to allow the same sample of blood to be analysed at each given time point. All TEG6S assays were performed in automatically loaded microfluidic cartridges. Patient characteristics, reason for ICU admission and/or type of surgery was recorded and stored anonymously on secure databases. Conventional coagulation tests (prothrombin time, international normalised ratio, activated partial thromboplastin time, fibrinogen level and platelet count) taken on the day of sampling were also recorded for the interdevice group. Volunteer age and gender information was also recorded and stored in a deidentified manner on a secure database.

### Statistical analysis

Interdevice agreements were estimated using Lin’s concordance correlation coefficient, (Lin’s CC), and further explored using intraclass correlation coefficient (ICC) and reduced major axis regression)^10–13^. Reduced major axis regression allows disagreement to be separated into fixed and proportional components^14^. Readings from two devices may differ by a consistent amount across the magnitude of the readings (fixed bias) or differ by a changing amount across magnitude (proportional bias) - in the latter case the slope of the reduced major axis regression line will differ from that of the line of perfect agreement (slope = 1)^15^. We used the Landis and Koch Benchmark Scale to describe interdevice agreement based on observed Lin’s CC: “Poor” (Lin’s CC: 0), “Slight” (Lin’s CC: 0.01–0.20), “Fair” (Lin’s CC: 0.21–0.40), “Moderate” (Lin’s CC: 0.41–0.60), “Substantial” (Lin’s CC: 0.61–0.80), and “Almost Perfect” (Lin’s CC: 0.81–1.00)^16^. Due to the absence of pre-existing information regarding anticipated levels of interdevice agreement, the appropriate sample size was determined based on pragmatic considerations of having 0.8 power of being able to reject a null hypothesis of moderate agreement in favour of an alternative of almost perfect agreement for a generic outcome, assuming alpha 0.05. Using the method described by Walter et al., wherein, assuming Type I error level alpha of 0.05, two independent rating devices, and the null hypothesis of interdevice agreement of 0.5 to be rejected, the sample size of 22 patients would yield 0.8 power to reliably observe excellent agreement of 0.8 or above^17^. We recruited 25 patients to account for potential non-evaluable data. No correction for multiplicity of outcomes was made due to the exploratory nature of the study. For time point test-retest reliability analysis we used a variance component model implemented as random effect linear regression model with individual volunteer participants used as random effects. The intraclass correlation coefficient produced by the model separates the reliability into time and operator/device components. Higher values of intraclass correlation coefficient are indicative of higher re-test reliability.

Statistical analysis was performed using commercial statistical software STATA/IC v.13IC® (StataCorp, College Station, TX, USA), using a p value of 0.05 to indicate the threshold for statistical significance. Indeterminate or missing data when secondary to true values being lower than what was recordable were assigned a value of zero, and when secondary to true values being higher than recordable were assigned the maximal value for that parameter. Missing data due to failed sample analysis from the TEG6S were omitted and left blank.

## Results

### Primary outcome: interdevice reliability

A total of 25 ICU patients were enrolled in the interdevice component of the study. Two TEG6S devices were used, and all analyses were performed by a single operator. Each sample of blood was performed once on each device. There was a total of 50 tests. Patient characteristics from tests performed included 16 (64%) males and 9 (36%) females. The median (interquartile range [IQR]) age was 61 years (23:86). The admission ICU diagnoses of these patients were: post-cardiac surgery (24%), decompensated liver disease (16%), post-liver transplantation (16%), post-general surgery (8%), neurological injury (16%), other critical illness (12%). During the testing period, there were no patients on therapeutic anticoagulation therapy including novel oral anticoagulants (direct thrombin and factor Xa inhibitors), warfarin, or heparin. There were no violations of the study protocol. Conventional coagulation tests taken on the day of sampling fell within normal reference ranges, revealing no gross coagulation abnormalities (Table 1).

**Table 1:** Conventional coagulation test results for patients used for interdevice testing (n = 25).

Coagulation Test	Median (IQR) Normal reference range
Prothrombin time (sec)	13 (11–15) 11–15
International normalised ratio	1.2 (1.1–1.4)
Activated partial thromboplastin time (sec)	30 (25–38) 22–41
Fibrinogen (g/L)	2.7 (2.1–4.4) 2.0–4.0
Platelets (x10^9^ cell/L)	129 (62–186) 150–400

Almost perfect interdevice agreement was observed, pervasive across all TEG parameters. The Lin’s concordance correlation coefficients (95% CI, slope, intercept) were CK R-time: 0.96 (0.92–0.99, 0.88, 0.57); K-time: 0.93 (0.87–0.98, 1.07, 0.00); Alpha Angle: 0.87 (0.78–0.96, 1.20, −14.10); Maximum Amplitude (MA): 0.99 (0.98–0.99, 1.02, -1.38); Clot Lysis (LY30%): 0.89 (0.82–0.97, 1.20, 0.07). All other variables assessed for this component of the study are presented in Table 2.

**Table 2:** Interdevice reliability of thromboelastograph parameters between two TEG6S devices.

Parameter	Lin’s Concordance Coefficient (95% CI)	Reduced Major Axis Intercept	Slope
CK R time	0.96 (0.93–0.99)	0.57	0.88
CK K time	0.93 (0.87–0.98)	0.01	1.07
CK alpha	0.87 (0.78–0.96)	−14.10	1.20
CK MA	0.99 (0.98–1.00)	−1.38	1.03
CK LY30	0.89 (0.82–0.97)	0.07	1.20
HK R time	0.95 (0.92–0.99)	0.33	0.94
HK K time	0.99 (0.98–1.00)	0.04	0.99
HK alpha	0.95 (0.91–0.99)	−9.90	1.13
HK MA	1.00 (0.99–1.00)	0.19	1.00
RT R time	0.94 (0.89–0.99)	−0.03	0.95
RT K time	1.00 (1.00–1.00)	0.03	0.95
RT alpha angle	0.99 (0.99–1.00)	2.00	0.98
RT MA	1.00 (1.00–1.00)	0.32	1.00
RT LY30	0.95 (0.92–0.98)	0.02	0.79
RT ACT	0.96 (0.93–0.99)	−2.96	0.94
FF MA	0.99 (0.98–1.00)	−0.55	1.06
FF FLEV	0.99 (0.98–1.00)	−13.60	1.07

Abbreviations: CK: standard kaolin; HK: standard kaolin with heparinase; RT: rapid thromboelastograph; R: reaction; K: kinetic; MA: maximum amplitude, LY: amplitude at 30 minutes; ACT: activated clotting time; FF: functional fibrinogen; FLEV: functional fibrinogen level. Reduced major axis slope different to 1 is indicative of the presence of proportional bias; the combination of the slope close to 1 and intercept different from 0 is indicative of the presence of fixed bias.

### Exploratory outcomes: time point reliability

Test re-test reliability analysis was performed on 15 participants (5 volunteers, 5 patients undergoing major abdominal surgery, and 5 ICU patients). The median (IQR) age was 54 years (22:71). Seven (47%) of these participants were male. High reliability across time points was observed for most parameters of interest, with the exception of CK K time and alpha angle, which demonstrated moderate reliability. Random-effects model intra-class correlation coefficients (95% CI) were CK R-time: 0.81 (0.66–0.91, p = 0.000); K-time: 0.67 (0.48–0.85, p = 0.000); Alpha Angle: 0.61 (0.39–0.80, p = 0.000), MA: 0.96 (0.91–0.98; p = 0.000), LY30%: 0.80 (0.61–0.91; p = 0.002) (Table 3).

**Table 3:** Time point (test re-test) reliability across thromboelastograph parameters.

Parameter	Intra-class correlation coefficient (95% CI)
CK R time	0.81 (0.66–0.91)
CK K time	0.69 (0.48–0.85)
CK alpha	0.61 (0.39–0.80)
CK MA	0.96 (0.91–0.98)
CK LY30	0.80 (0.61–0.91)
HK R time	0.70 (0.50–0.86)
HK K time	0.83 (0.68–0.92)
HK alpha	0.56 (0.34–0.77)
HK MA	0.96 (0.93–0.98)
RT R time	0.73 (0.54–0.87)
RT K time	0.96 (0.91–0.98)
RT alpha	0.96 (0.92–0.98)
RT MA	0.97 (0.94–0.99)
RT LY30	0.96 (0.91–0.98)
RT ACT	0.73 (0.54–0.87)
FF MA	0.97 (0.94–0.99)
FF FLEV	0.97 (0.94–0.99)

Abbreviations: CK: standard kaolin; HK: standard kaolin with heparinase; RT: rapid thromboelastograph; R: reaction; K: kinetic; MA: maximum amplitude, LY: amplitude at 30 minutes; ACT: activated clotting time; FF: functional fibrinogen; FLEV: functional fibrinogen level.

## Discussion

We observed high reliability between TEG6S results when sample analysis was performed using different TEG6S devices. This was pervasive across all TEG6S parameters, with no clinically significant differences noted. We also observed high reliability between results for most parameters when sample analysis was performed at different lengths of time.

This study is the first study to use a dedicated methodology to separately demonstrate high interdevice and time point reliability of the TEG6S. We have reinforced the findings of a recent validation study that concluded that the TEG6S had high precision within devices^6^. In addition, we have expanded the validity of the TEG6S to include not only patients undergoing cardiovascular surgery or percutaneous coronary intervention, but also a wider variety of critically ill patients (e.g. liver transplant and general surgical patients, and patients with decompensated liver failure and/or neurological injury) in whom TEG might be used to guide clinical decision making. Similarly, while it has previously been shown that results of TEG6S testing can be relied upon until up to 4 hours after sample collection in healthy volunteers^4^, we have further extended this finding to a range of up to 180 minutes. Finally, this study provides additional information with which to interpret novel research on the reliability of the TEG6S in non-standard clinical situations such as extremes of motion and temperature as might occur in pre-hospital or military environments^18–20^.

**Figure 1:**
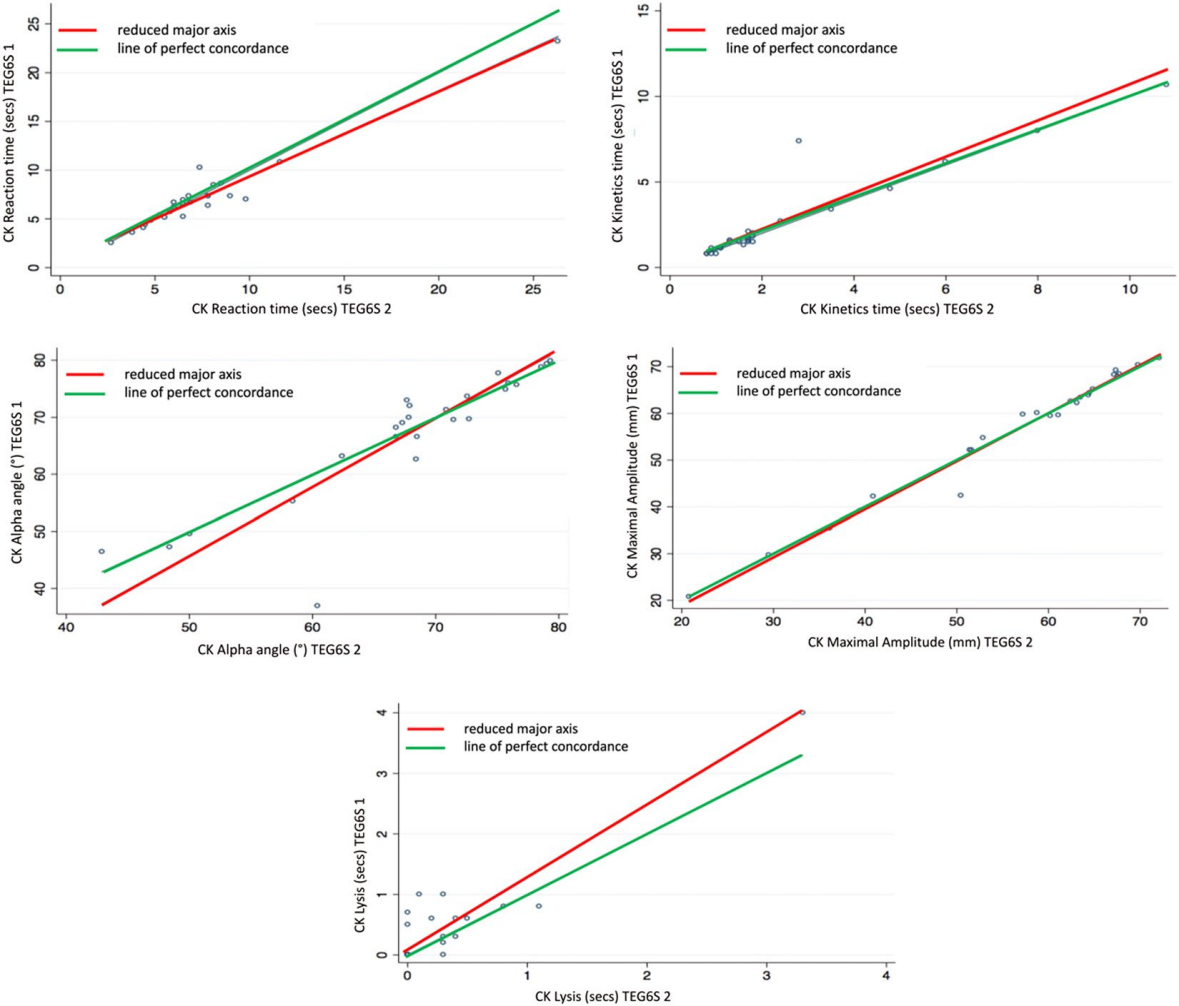
Citrated kaolin (CK): Interdevice agreement of reaction time, kinetics time, alpha angle, maximal amplitude and lysis.

**Figure 2:**
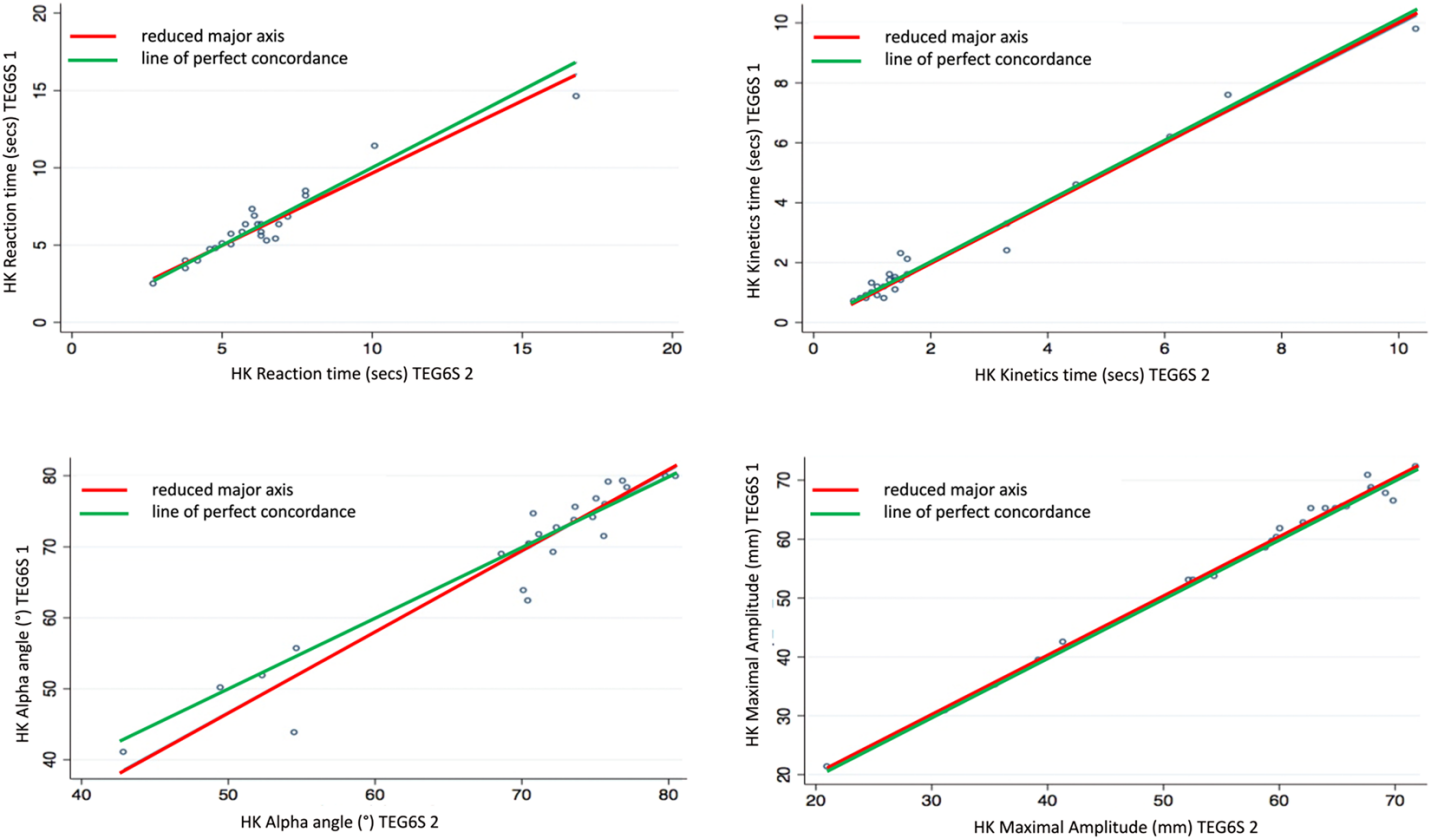
Heparinase kaolin (HK): Interdevice agreement of reaction time, kinetics time, alpha angle and maximal amplitude.

**Figure 3:**
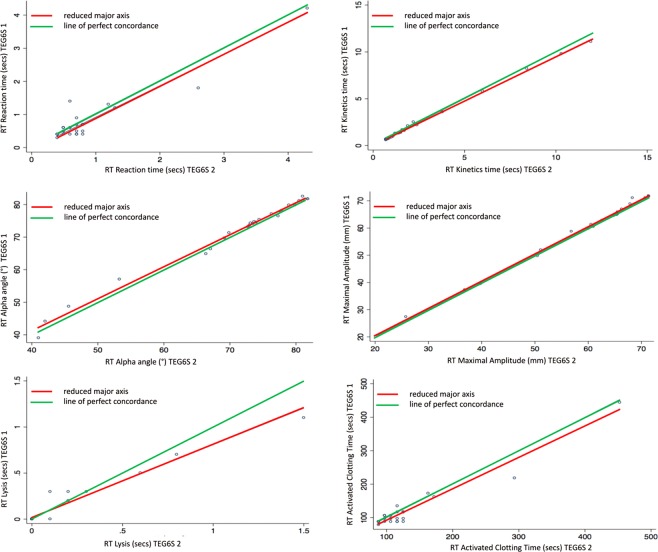
Rapid thromboelastograph (RT): Interdevice agreement of reaction time, kinetics time, alpha angle, maximal amplitude, lysis and activated clotting time.

**Figure 4:**
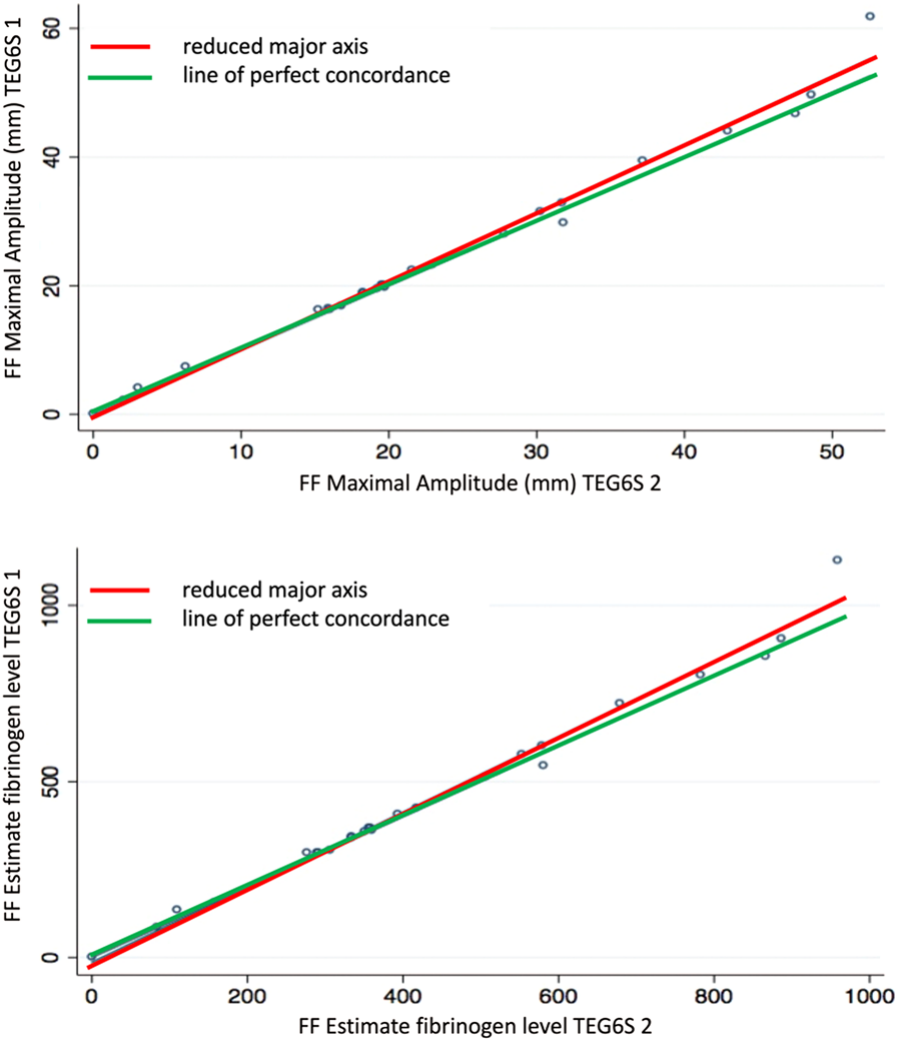
Functional fibrinogen (FF): Interdevice agreement of FF-derived maximal amplitude and estimated fibrinogen level.

The main component of this study was interdevice reliability. This component had the largest sample size and simplest method and analysis within this study. We showed that the TEG6S was able to provide clear evidence that demonstrates high agreement regardless of which specific device the samples are analysed on. This is best represented graphically in Figs. 1–4. This study component is the largest independent, prospective validation study to date comparing TEG6S reliability between devices in a real-world clinical setting. Our results suggest that clinicians and researchers can be confident interpreting results from different devices. Whilst our methods preclude comparing samples analysed at time points at extremes of sampling (0 min vs 180 min), our data provides evidence that no significant differences in TEG results were observed across these time points. Our data suggests the possibility that samples may be collected as part of routine phlebotomy rounds, and analysed in a laboratory at variable lengths of time after collection with minimal influence over the result, offering greater potential for TEG to be incorporated into standard care of ward patients.

Our study has independently validated TEG using two dedicated study arms to assess performance across devices and time points. Whilst other studies have looked at TEG6S performance, these have extrapolated results from a single data set^6^. We implemented a pragmatic study design and sampling technique, using broad volunteer and patient groups, reflecting real-world, typical clinical practice. Our study has practical implications, by focusing our assessment of factors that commonly influence sampling in clinical practice: different machines and different times post sampling. Our study has some limitations. A key limitation is small sample size used for our test-retest outcomes. We acknowledge this component of the study contained 15 participants, however we used robust and appropriate statistics to account for these numbers, in addition to acknowledging this as an exploratory, secondary outcome of the study. Additionally, we enrolled a number of patients from a single centre, however by doing this, we were able to examine TEG6S performance across a wide variety of patient groups (critically ill intensive care patients and intra-operative surgical patients). Conducting the study within a single-centre was beneficial for ensuring consistent methodology and adherence to study protocol. We conducted a validation study using two TEG devices and acknowledge that the use of more than two devices may have increased the reliability of our results, however we believe the number of devices tested were sufficient to answer our primary research question and achieve our study aims. Our study was non-interventional and was not powered to investigate TEG6S as a predictor of clinical outcomes (e.g. bleeding) however, this was not the intention of this study, being purely observational for validating a new and existing technology.

## Conclusions

By conducting a prospective observational study on volunteers, surgical and ICU patients, we observed that TEG6S results demonstrate almost perfect agreement when samples are analysed on different TEG6S devices moderate-to-high reliability when analysed at varying times after collection. This stability across devices and time points was pervasive across all TEG6S parameters. To our knowledge, this is the first prospective, independent, observational study performed validating the TEG6S viscoelastic device across these domains. Our results suggest that TEG6S results obtained on different machines can be compared with reasonable confidence, and provides preliminary evidence that results obtained after different time points could also be compared. This has positive implications for the utility of the TEG6S device both as a clinical and research platform, suggesting it provides reliable results under constraints of several common real-world conditions. These results have important implications for the use of TEG6S technology, particularly with a view to incorporate TEG6S into standard care for ward patients and comparing samples across a range of clinical practice and research projects.

## Data Availability

Data can be made available upon request to the corresponding author.
